# Spot the Difference—Development of a Syndrome Based Protein Microarray for Specific Serological Detection of Multiple Flavivirus Infections in Travelers

**DOI:** 10.1371/journal.pntd.0003580

**Published:** 2015-03-13

**Authors:** Natalie B. Cleton, Gert-Jan Godeke, Johan Reimerink, Mathias F. Beersma, H. Rogier van Doorn, Leticia Franco, Marco Goeijenbier, Miguel A. Jimenez-Clavero, Barbara W. Johnson, Matthias Niedrig, Anna Papa, Vittorio Sambri, Adriana Tami, Zoraida I. Velasco-Salas, Marion P. G. Koopmans, Chantal B. E. M. Reusken

**Affiliations:** 1 Erasmus Medical Centre, Viroscience Department, Rotterdam, The Netherlands; 2 National Institute for Public Health and Environment, Center for Infectious Diseases Research and Screening, Bilthoven, The Netherlands; 3 Oxford University Clinical Research Unit, Hospital for Tropical Diseases, Ho Chi Minh City, Vietnam; 4 Centre for Tropical Medicine, Nuffield Department of Medicine, University of Oxford, Oxford, United Kingdom; 5 Arbovirus and Imported Viral Diseases Laboratory, National Centre for Microbiology, Instituto de Salud Carlos III, Majadahonda, Madrid, Spain; 6 Centro de Investigación en Sanidad Animal (CISA), Instituto Nacional de Investigación y Tecnología Agraria y Alimentaria (INIA), Ctra Algete- El Casar, Valdeolmos, Madrid, Spain; 7 Diagnostic & Reference Laboratory, Arboviral Diseases Branch, Division of Vector-Borne Diseases (DVBD), Centers for Disease Control and Prevention (CDC), Fort Collins, Colorado, United States of America; 8 Centre for Biological Threats and Special Pathogens, Robert Koch-Institut, Berlin, Germany; 9 National Reference Centre for Arboviruses and Hemorrhagic Fever Viruses, Department of Microbiology, Medical School, Aristotle University of Thessaloniki, Thessaloniki, Greece; 10 DIMES—University of Bologna, Unit of Microbiology, Bologna, Italy; 11 The Greater Romagna Area Hub Laboratory, Pievesestina, Italy; 12 Department of Medical Microbiology, University of Groningen, University Medical Centre Groningen, Groningen, The Netherlands; 13 Departamento de Parasitología, Facultad de Ciencias de la Salud, Universidad de Carabobo, Valencia, Venezuela; 14 Departamento de Biología, Facultad Experimental de Ciencia y Tecnología, Universidad de Carabobo, Valencia, Venezuela; Mahidol University, THAILAND

## Abstract

**Background:**

The family *Flaviviridae*, genus *Flavivirus*, holds many of the world’s most prevalent arboviral diseases that are also considered the most important travel related arboviral infections. In most cases, flavivirus diagnosis in travelers is primarily based on serology as viremia is often low and typically has already been reduced to undetectable levels when symptoms set in and patients seek medical attention. Serological differentiation between flaviviruses and the false-positive results caused by vaccination and cross-reactivity among the different species, are problematic for surveillance and diagnostics of flaviviruses. Their partially overlapping geographic distribution and symptoms, combined with increase in travel, and preexisting antibodies due to flavivirus vaccinations, expand the need for rapid and reliable multiplex diagnostic tests to supplement currently used methods.

**Goal:**

We describe the development of a multiplex serological protein microarray using recombinant NS1 proteins for detection of medically important viruses within the genus *Flavivirus*. Sera from clinical flavivirus patients were used for primary development of the protein microarray.

**Results:**

Results show a high IgG and IgM sensitivity and specificity for individual NS1 antigens, and limited cross reactivity, even within serocomplexes. In addition, the serology based on this array allows for discrimination between infection and vaccination response for JEV vaccine, and no cross-reactivity with TBEV and YFV vaccine induced antibodies when testing for antibodies to other flaviviruses.

**Conclusion:**

Based on these data, multiplex NS1-based protein microarray is a promising tool for surveillance and diagnosis of flaviviruses.

## Author Summary

The number of international travelers has increased dramatically in recent decades. This has contributed to the increase in infectious diseases in travelers which are not present in their countries of origin and so may cause a threat to the public health. Viruses transmitted by biting insects (vector-borne viruses) are an important group within these travel-related diseases. They are found across the world and can cause debilitating and life-threatening symptoms, like inflammation of the brain or excessive bleeding. Many of these diseases are difficult to distinguish from each other. They cause comparable symptoms and are genetically closely related. Testing for long lists of diseases is time consuming and expensive. Here we develop a novel testing tool that allows doctors and researchers to test for multiple viruses with just one test. The method, which uses a specific part of the virus that makes distinguishing between infections with these closely related viruses possible, requires only one drop of blood. This allows us to test for multiple viruses simultaneously with the same amount of blood previously used to test for only one virus, while distinguishing between genetically closely related viruses.

## Introduction

The family *Flaviviridae*, genus *Flavivirus*, holds many of the world’s most prevalent arboviral diseases that are also considered the most important travel related arboviral infections.[1] As the geographic distribution and symptoms caused by these viruses overlap, detection requires differential diagnostic algorithms that include multiple flaviviruses.[2] Increase in travel expands the need for rapid and reliable multiplex diagnostic tests in non-endemic countries to supplement currently used methods.[3, 4]

Flaviviruses are single stranded enveloped viruses with an RNA genome of about 11 kb length. The genome is composed of three structural (Envelope, Capsid and Precursor-membrane) and seven non-structural proteins (NS1, NS2A, NS2B, NS3, NS4A, NS4B and NS5).[5] Diagnosis is primarily based on serology through detection of IgM and IgG antibodies, as viremia typically has been reduced to undetectable levels when symptoms set in and patients seek medical attention.[5–7]

**Fig 1:**
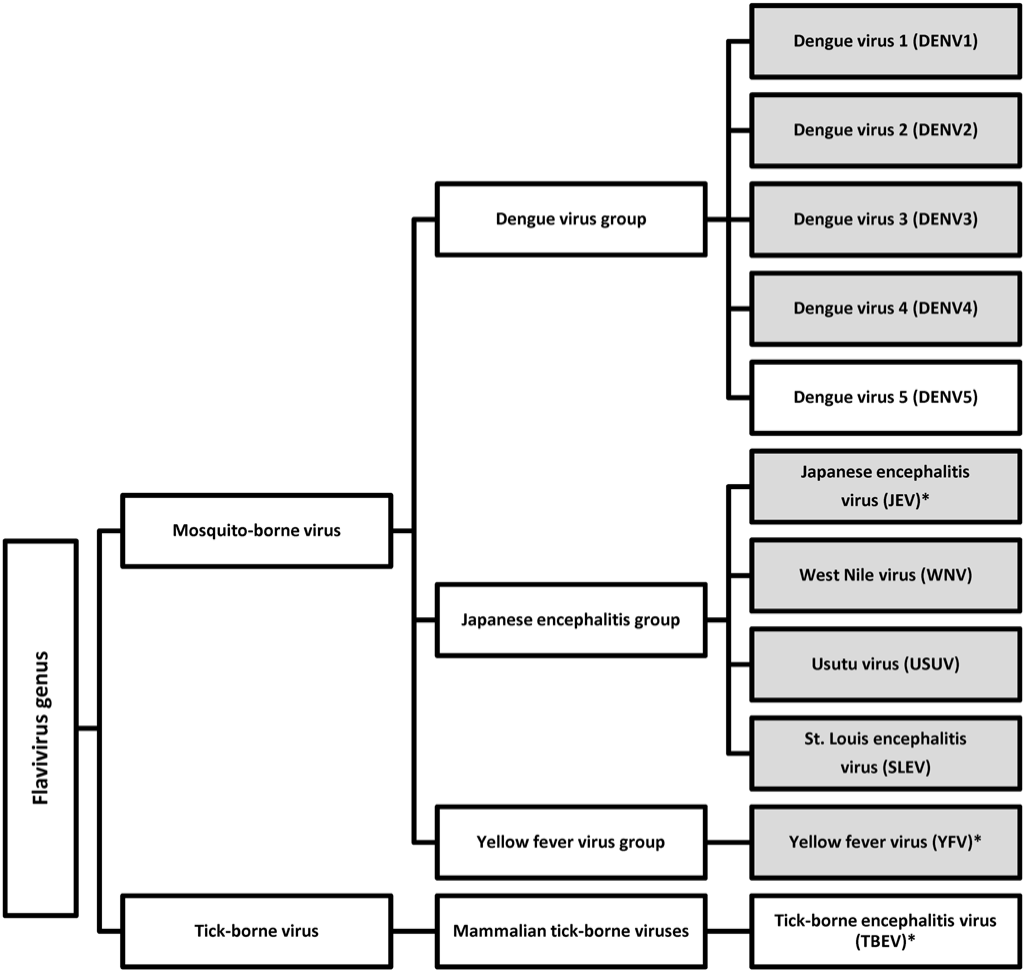
The serogroup classification of the *Flavivirus* genus of arboviruses used. Shaded boxes indicate antigens and antibodies used in this validation. Viruses with an * indicate that human vaccines are available for this virus.

The genus is divided into serocomplexes that are distinguished based on neutralizing antibody reactivity (Fig. 1). The amino acid homology of the envelope (E) protein (which is the immunodominant antigen for neutralizing antibody assays) ranges from 40–50% between serocomplexes and 70–80% for virus species within a serocomplex.[5, 8] Antibodies to flaviviruses are known to cross-react extensively within, and to a certain extent between, serocomplexes when using traditional antibody assays.[9–12] Cross-reactivity occurs also if patients have been vaccinated against flaviviruses such as yellow fever virus (YFV), tick-borne encephalitis virus (TBEV) and/or Japanese encephalitis virus (JEV) or after secondary infection with a different flavivirus.[9, 10, 13]

To overcome flavivirus cross-reactivity in diagnostics the use of recombinant antigens in ELISA is to be preferred over whole virus as it increases specificity.[14–16] Envelope, pre M and NS1 recombinant proteins are the most commonly used.[14–16] Of these, the NS1 has shown to be highly immunogenic and important in the development of non-neutralizing protective antibodies.[17, 18] NS1 is thought to contain more species specific epitopes than the envelope protein, although some cross-reactivity is seen between NS1 proteins.[19–21] NS1 in its natural conformation is thought to elicit a more specific immune response.[22, 23] The absence of NS1 proteins in inactivated JEV vaccines offers further potential for serological diagnosis through allowing differentiation between vaccinated and infected patients.[24] Thus, NS1 protein shows potential to use in serological differentiation between flavivirus infections.[25, 26]

To enable fast, syndrome based laboratory testing that focuses on multiple rather than individual viruses, we developed a protein microarray, using recombinant NS1 proteins, as a serological test for medically important viruses within the *Flavivirus* genus.

## Materials and Methods

### Samples

Sera from anonymized patients were used for primary development of the protein microarray. Patients were diagnosed according to international accepted criteria combining clinical symptoms, epidemiological data, and standard serological methods (ELISA, IFA) and laboratory confirmed by either VNT or PCR with the exception of 10 patients suspected of JEV. Information on each patient group used is presented in Table 1.

**Table 1:** Overview serum collection used for flavivirus microarray development.

Virus species*	County of origin	Number of samples	Days post onset symptoms	PCR confirmed	Virus neutralization confirmed (VNT/PRNT)	Serology (ELISA/IFA/ Luminex)
DENV1–2	Vietnam: Hospital for Tropical Diseases, Ho Chi Minh City, Vietnam	19	Hospitalized patients 2–7 days post onset symptoms	19/19	0/19	19/19
DENV1–4	Venezuela: Carabobo University, Faculty of Science and Technology, Department of Biology, Venezuela	12	3–21 days post onset symptoms	12/12	0/12	12/12
DENV1–3	Spain: National Centre for Microbiology. Instituto de Salud Carlos III.,Madrid, Spain	27	1–17 days post onset symptoms with travel history	27/27 (PCR or NS1-capture)	0/27	27/27
WNV	Greece: Department of Microbiology, Medical School, Aristotle University of Thessaloniki, Greece	7	9–23 days post onset symptoms	0/7	7/7	7/7
WNV	Netherlands: National Institute for Public Health and Environment, The Netherlands	5	5–21 days post onset symptoms with travel history	0/5	5/5	5/5
2xWNV; 1x SLEV; 1x YFV-vac	USA: US Centers for Disease Control and Prevention, Division of Vector-Borne Diseases, Arbovirus diagnostic and reference laboratory	4	Samples were part of the CDC 2011 reference panel for WNV serology	0/4	4/4	4/4
JEV	Vietnam: Hospital for Tropical Diseases, Ho Chi Minh City, Vietnam	10	From hospilized patients with acute encephalitis 6–18 days post onset symptoms	0/10	0/10	10/10: serially tested by two independent tests (ELISA and IFA) at two independent laboratories**
1x JEV; 1x YFV	Netherlands: National Institute for Public Health and Environment & Erasmus Medical Centre, The Netherlands	2	From hospitalized clinical patients 5–10 post onset symptoms with travel history	1(YFV)/2	2/2	2/2
1x pooled USUV	Centro de Investigación en Sanidad Animal, Madrid, Spain	1	Pooled rabbit sample 14 days post infection	1 /1	1/1	No tests available
2x human USUV	DIMES—University of Bologna, Unit of Microbiology, Italy	2	The only two human encephalitis cases reported in Europe	0/2	2/2	No tests available
Base-line group	The Netherlands: National Institute for Public Health and Environment	82	Dutch blood donors with unknown travel history and vaccination history	0/82	0/85	82/82: without detectable antibodies to WNV, DENV or TBEV
Vaccinated group	The Netherlands: National Institute for Public Health and Environment & Erasmus Medical Centre, The Netherlands and Germany: Centre for Biological Threats and Special Pathogens, Robert Koch-Institut, Germany	23	Vaccinated individuals with proven YFV, TBEV and/or JEV IgG titers	0/23	19/23	23/23
1x pooled JEV/DENV negative control; 1x pooled DENV1–4 positive control; 1x pooled post-JEV-vac	UK: NIBSC National Institute for Biological Standards and Control, UK	3	International reference samples: reference number #01/184, #01/186, #01/182	3/3	3/3	3/3

* DENV1–4 = Dengue virus serotype 1 to 4; JEV = Japanese encephalitis virus; SLEV = St. Louis encephalitis virus; TBEV-vac = Tick-borne encephalitis vaccinated; USUV = Usutu virus; WNV = West Nile virus; YFV = Yellow fever virus; YFV-vac = Yellow fever virus vaccinated;

** Hospital for Tropical Diseases, Ho Chi Minh City, Vietnam and National Institute for Public Health and Environment, the Netherlands

### Protein production

Custom-made NS1 proteins produced in human embryonic kidney 293 (HEK293) cells to ensure proper folding, glycosylation and dimerization were used (Immune Technology Inc., New York, NY, USA). A V5-epitope and Histag were added to the C-terminus for protein quantification and filtration. Proteins were expressed for Dengue virus 1 (genbank:FJ687432.1), Dengue virus 2 (genbank:FJ744720.1), Dengue virus 3, (genbank:FJ744738.1), Dengue virus 4 (genbank:EU854300.1), Japanese encephalitis virus (genbank:NC_001437.1), St. Louis encephalitis virus (genbank:ACB58159.1), Yellow fever virus (genbank:JN620362.1) and West Nile virus (genbank:EU081844.1)

Usutu virus NS1 (genbank:NC006551.1) was produced in-house in a HEK293 cell-line. The NS1 gene was produced by Genscript (NJ, USA) with an additional V5-epitope and Histag on the C-terminus and cloned into a pcDNA-DEST40 vector (Invitrogen, Thermo Fisher Scientific, MA, USA) that contained a neomycine resistance gene. The vector which contained the NS1 gene was transfected into HEK293 cells. Neomycine resistant clones were selected and tested for protein expression by immune fluorescent assay using anti-V5 monoclonal antibody. Selected clones were grown in flasks and secreted NS1 protein into the medium (Opti-MEM, Thermo Fisher Scientific, MA, USA). The secreted protein was purified from the medium by FPLC using a Ni-NTA column (Qiagen, CA, USA) according to the manufacturer’s instructions.

### Microarray slide preparation

NS1 antigens at concentrations of around 2mg/ml were mixed with protein arraying buffer (Maine manufacturing, GVS Group, Italy) and spotted in triplicate as a within-test control per pad. Antigens were spotted onto a nitrocellulose pad coated glass slide (Maine manufacturing, GVS Group, Italy) using a non-contact protein array spotter (PerkinElmer, Waltham, MA, USA) as previously described.[27] Per spot two drops of 333 pL of diluted protein were used. After printing, slides were placed in a drying chamber overnight and stored at room temperature until use.

### Protocol testing for IgG and IgM antibodies

Patient sera were tested on dried slides as previously described.[27] In short, slides were incubated in Blotto blocking-buffer (Thermo Fisher Scientific, MA, USA) for one hour at 37°C in an incubation chamber to reduce non-specific binding of serum. Serum was diluted in eight two-fold dilution steps (1:10 to 1:2560) in blotto supplemented with 0.1% Surfact-Amp (Thermo Fisher Scientific, MA, USA) and incubated for 1 hour at 37°C in a moist chamber. Incubation followed with an Fc-fragment specific IgG or Fc5μ-fragment specific IgM specific conjugate with a Cy5-fluorescent dye (Invitrogen, CA, USA) for one hour at 37°C. For IgM detection, serum was first depleted of IgG antibodies using Gullsorb (Meridian Bioscience, OH, USA) according to the manufacturer’s instructions. Between each incubation step, slides were washed three times with a protein array washing buffer (Thermo Fisher Scientifc, MA, USA). After final wash, slides were scanned with a Tecan scanner (Tecan Trading AG, Männedorf, Switzerland). A median fluorescence signal (measured at 647nm) for each of the triplet spots per antigen was determined by ScanArray Express 4.0.0.0001 supporting program (PerkinElmer, MA, USA) using an adaptive circle (diameter 80–200 μm). The fluorescent signal ranged from 0 to a maximum of 65,536 units. Results were imported in R for analysis.[28]

### Protein concentration optimization

Virus antigens were spotted in serial two-fold dilutions ranging from 1:2 to 1:16 for initial checkerboard titrations to determine optimum protein concentration as previously described.[27] Antigens were tested using serially diluted anti-V5-epitope monoclonal antibodies (Invitrogen, Thermo Fisher Scientific, MA, USA). Optimum protein concentrations were defined as those at which maximum fluorescent signal and overlapping s-curves were achieved for anti-V5-epitope monoclonal antibodies and were found to be around 0.5 mg/ml. To minimize batch-to-batch variations each batch was tested with a serial dilution of anti-V5 monoclonal antibodies. If a variation of more than 10% was found in reference to the initial test batch the slides were excluded. Day-to-day variations were monitored by including a positive and negative WHO DENV1–4 reference serum during each test round. If a dilution-step difference of more than one titer was detected results were excluded.[29]

### Analysis

A script was written in R[28] using additional package ‘drc’ version 2.3–7[30], as previously described.[27] The median fluorescent signals were converted into fitted dilution-s-curves per protein for each serum sample. Additional script was written that allowed titers to be calculated on the estimated s-curve at a given ROC calculated cut-off. Optimal signal cut-offs were determined by a log2 transformation of signals to further reduce variance caused by day-to-day and slide-to-slide variations. Optimal signal cut-offs were achieved by selecting the highest possible combination of sensitivity and specificity through ROC optimal curve calculations performed in GraphPad Prism.[31] Titers were defined as the highest serum dilution with a signal above the cut-off determined by ROC analysis. Heat maps were generated using an additional R package ‘stats’[28] and based on pairwise correlation between rows and columns. Mann-Whitney tests were employed to establish the significance of differences between groups.

### Accession numbers

Dengue virus 1 (genbank:FJ687432.1), Dengue virus 2 (genbank:FJ744720.1), Dengue virus 3, (genbank:FJ744738.1), Dengue virus 4 (genbank:EU854300.1), Japanese encephalitis virus (genbank:NC_001437.1), St. Louis encephalitis virus (genbank:ACB58159.1), Yellow fever virus (genbank:JN620362.1) and West Nile virus (genbank:EU081844.1), Usutu virus NS1 (genbank:NC006551.1).

## Results

### Sensitivity and specificity for individual antigens

**Fig 2:**
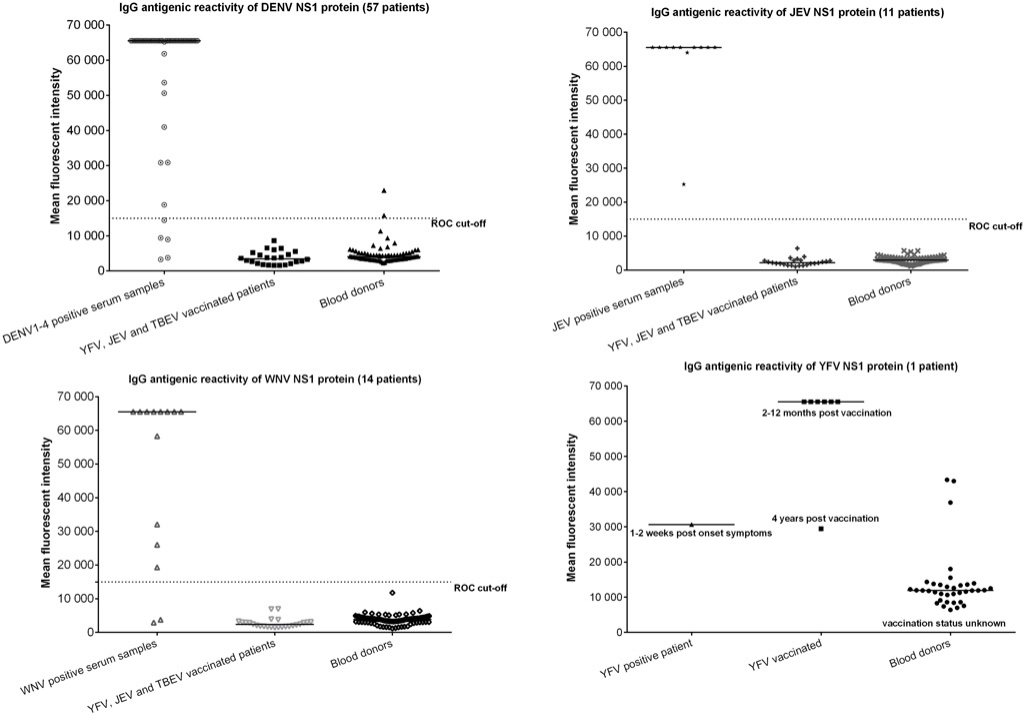
IgG fluorescent intensity (measured at 647nm) to flaviviruses in serum samples from clinical patients, persons vaccinated with YFV, JEV or TBEV and healthy blood donors in a 1:20 serum dilution. NS1 proteins were spotted in a 0,5mg/ml concentration. Y axis represents the fluorescent intensity. The median signal is depicted as a line. The dotted line represents the calculated cut-off by the ROC used to determine antibody titers.

**Fig 3:**
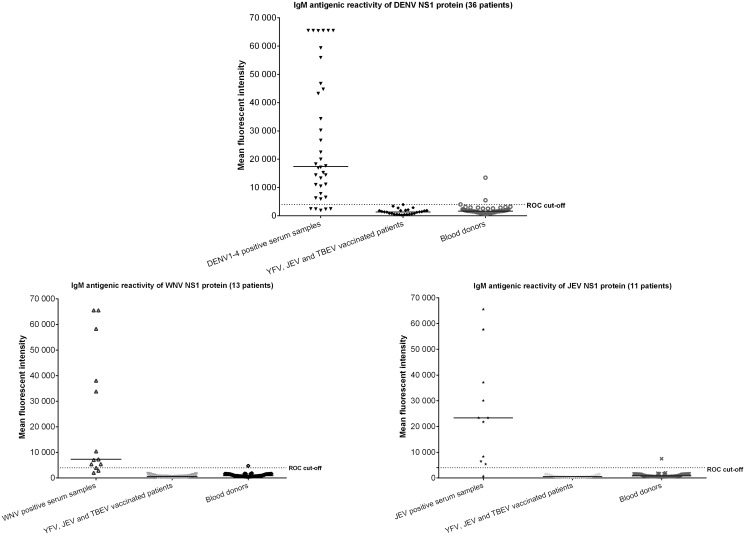
IgM fluorescent intensity (measured at 647nm) to flaviviruses in serum samples from clinical patients, persons vaccinated with YFV, JEV or TBEV and healthy blood donors in a 1:20 serum dilution. NS1 proteins were spotted in a 0,5mg/ml concentration. Y axis represents the fluorescent intensity. The median signal is depicted as a line. The dotted line represents the calculated cut-off by the ROC used to determine antibody titers.

The mean antigen reactivity by NS1 proteins in 1:10 to 1:80 start dilutions was high in homologous DENV, WNV, JEV, SLEV, YFV and Usutu virus (USUV) positive control sera and low in negative control sera and in sera from individuals vaccinated for JEV, TBEV or YFV (p<0.01) with the exception of YFV NS1 antigen with YFV vaccinees (Fig. 2, Fig. 3, and Table 2). Only some NS1 reactivity was observed in samples from blood donors for other antigens (1%).

**Table 2:** Median signal and 25–75% percentile for IgG and IgM per virus antigen test group.

Serum dilution	1:20 IgG		1:20 IgM			1:20 IgG		1:20 IgM	
VIRUS	Median	25–75% percentile	Median	25–75% percentile	CONTROL	Median	25–75% percentile	Median	25–75% percentile
Dengue	65,535	65,535	17,421	8,487–46,244	Vaccinated	3,440	2,105–5,070	1,387	536–1,876
					Blood donors	4,057	3,350–4,880	1,632	1,181–1,984
West Nile	65,535	24,350–65,535	7,311	4,678–48,174	Vaccinated	2,382	1,742–3,026	542	478–972
					Blood donors	3,861	3,025–4,471	817	591–1,456
Japanese encephalitis	65,535	65,535–65,535	23,364	65,04–37,139	Vaccinated	2,138	1,559–2,792	529	410–921
					Blood donors	2,945	2,368–3,563	806	568–1,527
Usutu	43,939	22,342–65,535	NA	NA	Vaccinated	2,804	1,961–3,101	ND	ND
					Blood donors	3,382	2,778–4,008	809	594–1,848
Yellow fever	30,680	30,680–30,680	NA	NA	Vaccinated	65,535	6,5535–6,5535	NA	NA
					Blood donors	11,925	9,497–13,576	3,163	1,346–4,160
St. Louis encephalitis	65,535	65,535–65,535	NA	NA	Vaccinated	ND	NA	NA	NA
					Blood donors	4,808	3,636–5,649	878	711–1,084

ND = Not done; NA = Not available; Vaccinated = Vaccinated for TBEV, YFV and/or JEV

At low serum dilutions, some patients showed antibody IgG reactivity to multiple antigens, and therefore ROC curves were calculated in multiple dilutions and the signals for the 1:20 dilutions were used for signal cut-off calculations. The 1:10 and 1:20 serum dilutions produced comparable results in sensitivity and specificity, but with significantly lower background for the 1:20 dilutions. At 1:40 serum dilutions, the sensitivity started to decrease.

Only 13 DENV positive patients (travelers) had known primary DENV infections with a PCR confirmed serotype (DENV1–3). All other patients with PCR confirmed DENV (serotype 1–4) were from DENV endemic countries and could not be confirmed as primary infections. As not all DENV infections were known to be primary, the highest signal to DENV1–4 NS1 was used for calculation of the DENV cut-offs. The optimal cut-off for all proteins was around a fluorescent signal of 15,000 for IgG and 4,000 for IgM, producing sensitivity and specificity of 86% to 100% (Table 3).

**Table 3:** Sensitivity and specificity for each virus antigen group with 95% CI.

Group	n samples	Sens IgG	Spec IgG (n82)	n samples	Sens IgM	Spec IgM (n80)
**DENV**	57	0.92 (0.93–0.98)	0.99 (0.95–1.00)	36	0.86 (0.71–0.95)	0.98 (0.93–1.00)
**WNV**	14	0.86 (0.57–0.98)	1.00 (0.97–1.00)	13	0.85 (0.55–0.98)	0.99 (0.95–1.00)
**JEV**	11	1.00 (0.72–1.00)	1.00 (0.97–1.00)	11	0.91 (0.59–1.00)	0.99 (0.95–1.00)

The number of positive samples are indicated in column ‘n samples’ and the number of negative samples are shown behind brackets in the ‘Spec’ column.

n samples = number of positive samples used; Sens = sensitivity; Spec = specificity

For USUV, SLEV and YFV only one or two positive patient samples were available so that proper ROC curves could not be calculated, but background signals were in the same range as for the other antigens (Table 2). Serum samples from YFV-vaccinees were strongly positive for YFV. Some blood donors had YFV signals above the cut-off, probably reflecting vaccination history (Fig. 2).

### Cross-reactivity

**Fig 4:**
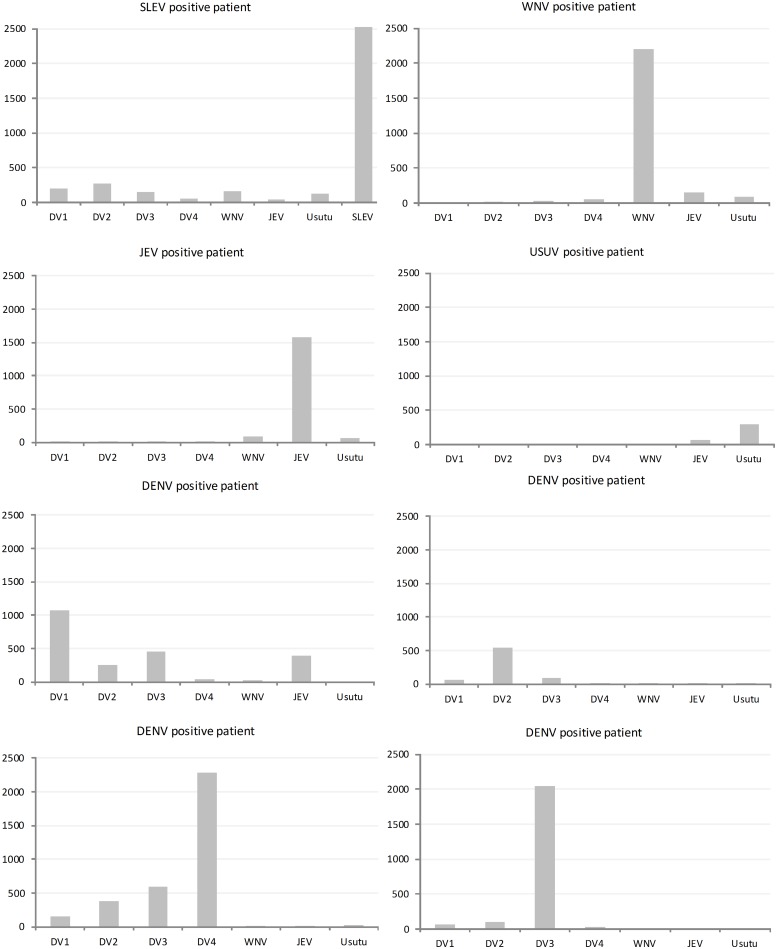
Representative examples of IgG antibody profiles of individual patients infected with JEV and DENV serocomplex viruses. Antibody profiles are presented as titers (y-axis) for each NS1-protein (x-axis).

**Fig 5:**
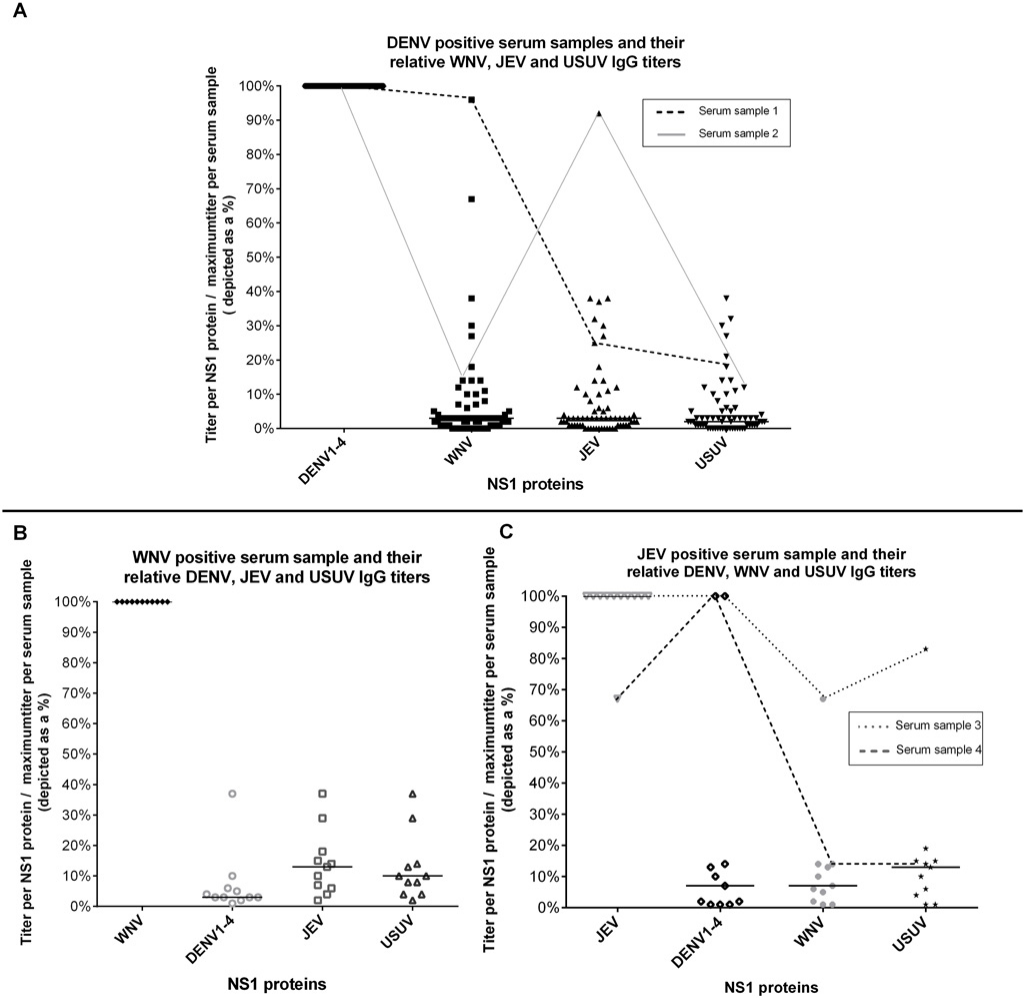
Analysis of cross-reactivity for microarray based flavivirus serology. To visualize the cross-reactivity seen in individual serum samples the maximum calculated titer per sample was set at 100% and all other signals were expressed as a percentage of the highest titer (0–100% on y-axis).

In order to study cross-reactivity within and between serocomplexes, serum samples were serially diluted and titers were calculated in R. Typical individual patient profiles are shown in Fig. 4. To quantify the degree of cross-reactivity, the ratio of the signal for each antigen to the maximum signal measured for that serum (typically the homologous antigen) was calculated (Fig. 5). With one exception for IgG (serum sample #4), all patients had the highest IgG and IgM reactivity with the homologous NS1 antigen. High level IgG reactivity to a second antigen was observed for two of the DENV patients (against WNV and JEV, respectively) and for 2 JEV patients (against DENV) (Fig. 5a). One serum sample from a JEV patient (serum sample 4) had a higher titer DENV NS1 in comparison to JEV NS1. For IgM, only homologous reactivity was observed.

### Serotype specific reactivity for DENV

**Fig 6:**
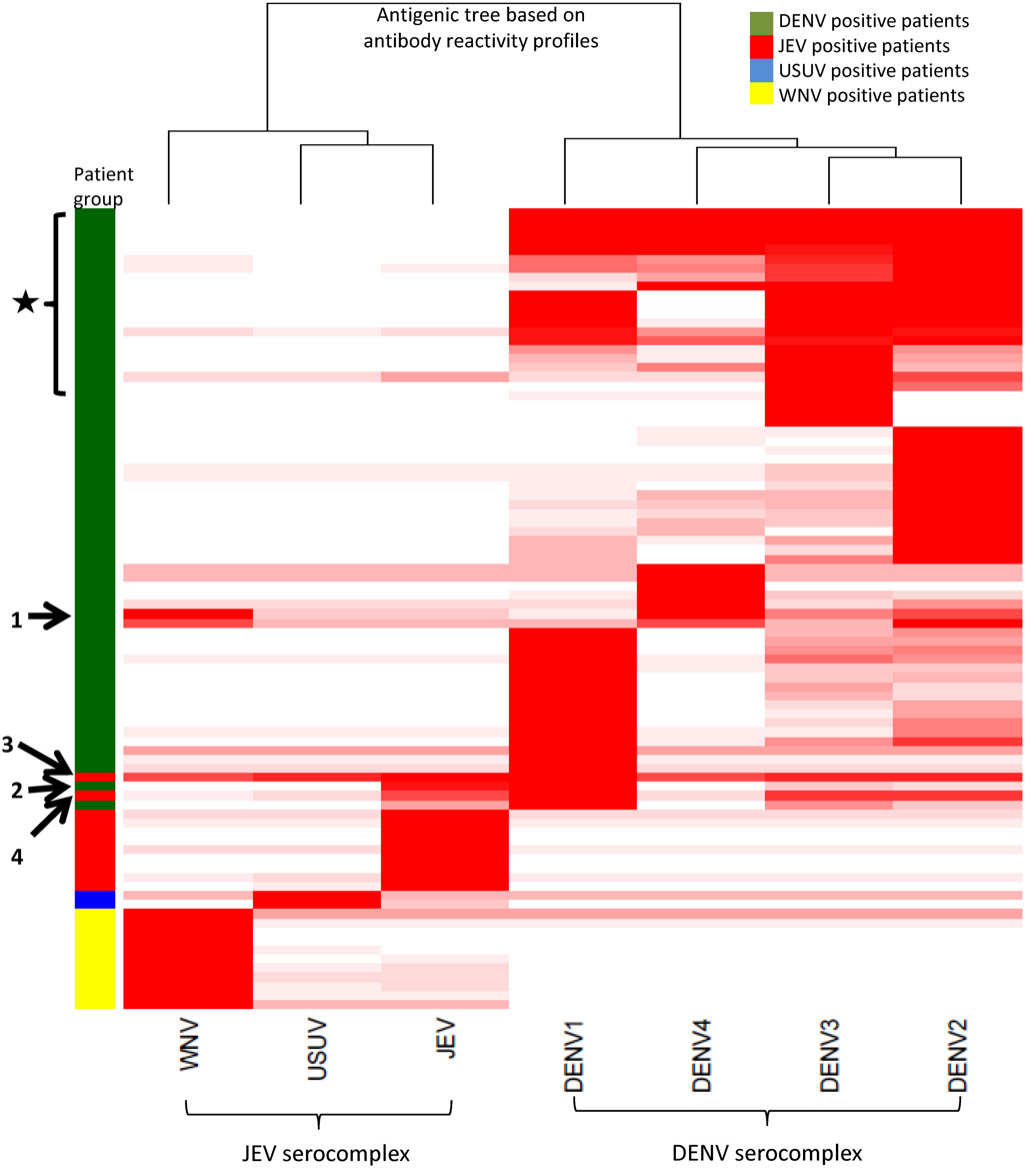
Heatmap of patient IgG antibody profiles. To visualize the overall cross-reactivity seen in individual serum samples the maximum calculated titer per sample was set at 100% and all other signals were expressed as a percentage of the highest titer and placed in a heatmap. White refers to a titer of 0% in reference to highest calculated titer per serum sample with a sliding scale to red which indicates a titer of 100% comparable to highest titer calculated. The numbers alongside the patient group column correspond to the serum samples shown in Fig. 4A and 4C. The star indicates a group of patients with high titers to multiple DENV NS1 antigens.

IgG profiles from individual patients were combined into a heatmap (Fig. 6) to confirm grouping according to exposure history. One group of patients (indicated by a star in the heatmap) showed high titers to multiple DENV serotypes. A larger group had highest titers to a single DENV serotype, suggesting serotype specificity of the antibody array results. As most patients were from different regions, the data were stratified for non-endemic (travelers) and multiple DENV endemic countries. This showed a significant difference in titers between groups (p<0.01) for IgG but not for IgM (p = 0.25) titers.

For 13 known primary DENV cases, the serotype had been determined by RT-PCR. All but one serum had highest IgG antibody levels to the infecting serotype, but IgM antibody reactivity was lower and less discriminatory.

## Discussion

We developed a first generation protein microarray for rapid, multiplex and virus-specific IgM and IgG tests for diagnosis of flavivirus infections that could differentiate between virus species, even within flavivirus serocomplexes, as well as between vaccinated and infected individuals, with the exception of YFV vaccinated individuals. Initial validation shows that this microarray can be used for flavivirus surveillance in travelers and potentially in regions with co-circulation of multiple flaviviruses. To establish this we first investigated the sensitivity of NS1 antigens to their homologous sera on a protein microarray. The results show good IgG and IgM sensitivity for both JEV and DENV serocomplex viruses. The sensitivity is comparable to current IFA and ELISA commercial kits. However, our sample selection was tested with a multitude of standard serological assays. Comparing our results to commercial ELISA or IFA kits should therefore be done with caution. Our results confirm that NS1 provides a good sensitive and specific antigen tool for serological diagnosis.

For DENV, the sensitivity of the IgM assay was lower than for IgG. There are several possible explanations for this finding. First, lower DENV IgM sensitivity may have resulted because five to ten days post onset of symptoms may be too early for detecting seroconversion in serum samples.[32] Evaluations of commercial flavivirus IgM diagnostics kits showed varying sensitivities ranging from 58% to 98%, partially due to sample timing.[32–34] Five days post onset of symptoms, around 50–80% of patients on average have detectable IgM antibodies. This increases to 99% 10 days post onset of symptoms.[35]

Second, a study of antibody reactivity to individual DENV proteins has found that the measured mean OD is lower for NS1 compared to E-protein with many samples clustering closer to the cut-off compared to E-protein antibodies.[18] This makes NS1 antibody detection more susceptible to timing of sample taking and detection limit of test used.

Third, patient-to-patient variation in antibody responses to individual viral antigens may cause discrepancies of test results. NS1 based protein assays could potentially pick up infections missed by prM-E based front-line serological tests.[20] Antibodies to envelope protein were detected in 91% of the DENV cases while NS1 antibodies were detected in 99%, indicating that NS1 has a higher sensitivity.[18] Two of the nine DENV IgM positive samples from travelers that tested negative on DENV IgG ELISA, which is based on the prM-E antigen, tested IgG positive on our microarray, further supporting this assumption.

Finally, a number of the DENV cases may have been secondary, tertiary or quaternary infections. Several of the samples originated from DENV hyperendemic countries and showed high titers to multiple DENV and other flaviviruses, further supporting this assumption. Literature shows that IgM antibody titers produced during a secondary DENV infection are absent or lower and produced during a shorter period. This reduces the sensitivity of IgM antibodies in non-primary infections significantly.[35] Despite the above limitations, the NS1-based protein microarray performed well. Ideally, further validation is needed with sequentially sampled patient sera, allowing evaluation optimization of timing of sampling during infection, in relation to cut-off chosen.

The biggest challenge of current flavivirus serodiagnostics is virus specific differentiation.[3, 4] We analyzed the signals produced towards all antigens per sample to test our protein microarray’s capability to achieve this. With three exceptions (probable secondary infections), clear differentiation of antibody responses to the homologous antigen (defined as the virus the patient was confirmed infected with) was found for most patients. This can be clearly seen in the heatmap (Fig. 6). This is a big advantage over currently available serological tests, for which particularly flavivirus vaccination causes extensive cross-reactivity, mainly for IgG.[9–12] Previous epitope analysis of DENV NS1 and envelope protein indicated that NS1 has more virus specific epitopes and thus could be used for more specific serological tests.[16, 19–22] Our results support this showing good specificity for the NS1-based protein microarray.

The cause of the outliers shown in Fig. 5 is unclear as they may be the result of cross-reactive antibodies or previous exposure. Cross-reactive antibodies to NS1 proteins have been detected in previous studies, but why they are seen in some patients and not others remains unclear.[20] The reactivity to multiple antigens more likely reflects differences in exposure history.[10, 36] This assumption is supported by the fact that most patients with reactivity to multiple antigens originated from countries where multiple flaviviruses are endemic (Vietnam and Venezuela). The IgG titers of these patients are log multiplications higher than singular reactive titers and their corresponding IgM titers, which is highly indicative for secondary and frequent flavivirus infections.[16] Distinguishing primary from secondary or multiple flavivirus infections is serologically difficult.[16] Further investigation into patients with known multiple flavivirus infections will be needed to further define the uses of our multiplex array testing in such patient populations.

While we show excellent discrimination in antibody responses to viruses within the JEV complex, this is less straightforward for DENV. The protein microarray IgG antibody reactivity to individual DENV NS1 antigens shows capability to distinguish serotypes (as can be seen in Fig. 6), but this is not seen to the same extent for IgM antibodies. This was surprising as flavivirus IgM envelope antibodies are thought to be more specific than IgG antibodies.[16] However, to what extent this can also be said for NS1 is unclear. Here, future work will focus on more extensive evaluation, for which well characterized patient cohorts are needed.

Finally, we looked at the ability to rule out false positive reactions due to vaccination. This was not possible for YFV vaccination when testing against YFV NS1. YFV vaccine is a live attenuated vaccine and causes mild infection resulting in NS1 antigen and antibody production.[37] We do, however, show that when using NS1 antigens the cross-reactivity between the YFV serocomplex and other serocomplexes is absent.[9, 10, 13] JEV vaccine currently on the European and American market is an inactivated whole virus vaccine based on JEV virions.[38] This production technique makes NS1 a good target for vaccination versus infection differentiation and is the basis for current surveillance programs in Japan and surrounding countries.[39] However, live-attenuated (non-recombinant) JEV SA-14–14–2 vaccine is currently in use in a number of countries in Asia and has recently been WHO prequalified. Its use nullifies the ability to differentiate vaccination from infection, reducing public health surveillance options. New vaccine methods using chimeric virus vaccines are in development and might provide new opportunities for vaccination and infection differentiation through NS1. In veterinary vaccine development, good practice ensures the ability for such discrimination through the development of marker vaccines according to the DIVA (Differentiating Infected from Vaccinated Animals) principle.[40] Public health challenges associated with inability to reliably provide patient diagnostics in some instances due to vaccination, highlight the need for introducing this principle to the human vaccine market.

### Conclusion

Serological differentiation between flaviviruses and the false-positive results caused by vaccination are a serious problem for surveillance and diagnostics of flaviviruses. Analysis of our NS1-based protein microarray results showed a high IgG and IgM sensitivity and specificity for individual antigens even within the same serocomplex, and limited cross reactivity. In addition, the serology based on this array allowed discrimination between infection and vaccination response for JEV vaccine, and no cross-reactivity with TBEV and YFV vaccine induced antibodies when testing for antibodies to other flaviviruses. Based on this data, our multiplex NS1-based protein microarray is a promising tool for surveillance and diagnosis of flaviviruses.
